# Intraabdominal hypertension in burn patients

**DOI:** 10.1186/cc14467

**Published:** 2015-03-16

**Authors:** A Mokline, I Rahmani, L Gharsallah, A Hachani, S Tlaili, R Hammouda, B Gasri, A Ksontini, AA Mesadi

**Affiliations:** 1 Trauma and Burn Centre of Tunis, Tunisia

## Introduction

Intra-abdominal hypertension (IAH) is frequent in the ICU and has been associated with adverse outcomes and worse prognosis. The purpose of our study was to assess risk factors for IAH and prognosis of major injured patients during burn resuscitation.

## Methods

Adult burned patients with a burn injury exceeding 20% of total body surface area, from 1 April to 30 November 2013, were included. IAP was measured when IAH was suspected, according to the Kron method via the Foley catheter. Monitoring of IAP was performed every 6 hours during 5 days until normalization.

## Results

Twenty patients were enrolled in the study. The mean age was 36 ± 13 years. There were 14 males and six females. The average TBSA was 44 ± 17%. Screening and monitoring of IAP were applied by: oliguria (42%), abdominal distension (31.5%) and gastrointestinal trouble (21%). IAH occurred between day 2 and day 3 after early burn resuscitation, respectively in 52% and 63%. IAH was observed in 69% of cases in patients admitted to the ICU with a delay of 1.6 days post burn injury. IAH was noted in 13 patients; of these, five patients developed an abdominal compartment syndrome. The mean IAP was 16 ± 7 mmHg. Patients were assigned into two groups: G1 (IAH+; *n *= 13) and G2 (IAH-; *n *= 7). Comparative study of the two groups shows that HIA increases significantly body weight gain within the first 5 days after injury: 8 kg for G1 versus 2 kg for G2 (*P *= 0.04), occurrence of ARDS (70% for G1 vs. 16.7% for G2, *P *= 0.02), respiratory failure (77% for G1 vs. 28.5% for G2, *P *= 0.06), shock (70% for G1 vs. 16.7% for G2, *P *0.02) and mortality (61.5% vs. 50%).

## Conclusion

IAH was frequent in early burn resuscitation of major injured patients. It seems to be associated with fluid overload in burns and contributes to organ damage.

